# Supplementary material for: Investigation on the Contents of Nε-carboxymethyllysine, Nε-carboxyethyllysine, and N-nitrosamines in Commercial Sausages on the Chinese Market
Source: Foods. 2023 Feb 7;12(4):724. doi: 10.3390/foods12040724 (PMC9955857; doi:10.3390/foods12040724)
Supplement: Supplementary file 1 [file foods-12-00724-s001.zip › foods-2105579-supplementary.pdf]

Table S1 MRM settings for CML, CEL, α-dicarbonyls, and NAs detection.

Compound	Precursor ion	Product ion	Fragmentor (v)	Collision Energy (ev)	Dwell Time (ms)
CML	205	130	60	10	50
		84*	60	12	50
d_4-CML	209	134	60	9	50
		88*	60	13	50
CEL	219	130	60	10	50
		84*	60	12	50
d_4-CEL	223	134	60	11	50
		88*	60	13	50
3-DG	235	199*	75	14	30
		171	75	27	30
DA	159	118	80	24	30
		77*	80	38	30
MGO	145	92	60	21	30
		77*	60	33	30
GO	131	77*	50	31	30
		55	50	50	30
NDMA	74	42		20	120
		44*		5	120
NDMA-d_6	80	48		7	120
		50*		3	120
NDEA	102	56		17	90
		85*		3	90
NDEA-d_{10}	112	62		15	90
		94*		5	90
NMEA	88	42		18	80
		71*		2	80
NDPA	130	43		10	70
		113*		0	70
NMOR	116	56		12	70
		86*		1	70
NPIP	114	97		7	90
		84*		7	90
NPYR	100	55*		7	70
		70		5	70
NPYR-d_8	108	62		7	70
		78*		5	70
NDBA	158	99		5	120
		141*		0	120
NDPHA	169	167		30	100
		168*		17	100

* Indicates ion pairs for quantification.

Table S2 The performance of NAs.

NAs	NDMA	NDEA	NMEA	NDPA	NPIP	NMOR	NPYR	NDBA	NDPhA
Coefficients (R^2)	0.9995	0.9994	0.9996	0.9997	0.9997	0.9996	0.9990	0.9996	0.9997
LOD (μg/kg)	0.5	0.2	0.4	0.4	0.1	0.5	0.9	0.1	0.02
LOQ (μg/kg)	1.6	0.6	1.3	1.3	0.3	1.6	2.8	0.3	0.06
Recovery (%)	85-104	101-113	77-93	82-85	89-97	85-98	111-116	75-76	87-99

Table S3 Moisture, fat, protein contents, pH, and TBARS values of commercial sausages in the Chinese market.

Samples	Moisture (%)	Fat (%)	Protein (%)	pH	TBARs (mg/kg)
Salami Al tartufo (F1)	38.16	26.93 ± 2.21	24.84 ± 0.63	5.03 ± 0.04	10.49 ± 0.85
Salami Toscano (F2)	39.29	25.89 ± 1.91	27.02 ± 0.15	5.63 ± 0.05	6.93 ± 0.63
Pepperoni (F3)	31.49	38.11 ± 0.50	23.83 ± 0.62	4.89 ± 0.01	4.31 ± 0.57
Basitides (F4)	31.77	31.03 ± 0.49	29.20 ± 0.97	5.34 ± 0.02	13.35 ± 0.43
Salami Ungherese (F5)	37.21	29.75 ± 1.67	23.86 ± 0.01	5.42 ± 0.06	1.98 ± 0.28
Salami Milano (F6)	31.78	33.19 ± 0.28	27.12 ± 1.09	4.97 ± 0.02	12.28 ± 0.27
Salami Napoli (F7)	32.30	34.41 ± 1.82	25.75 ± 1.64	4.95 ± 0.04	14.69 ± 1.53
TULIP Hungarian Salami (F8)	39.29	32.09 ± 1.32	20.52 ± 0.99	4.96 ± 0.03	1.44 ± 0.14
Palacios original chorizo (F9)	23.57	43.25 ± 0.66	24.07 ± 0.69	5.82 ± 0.02	1.65 ± 0.13

Average	30.00	34.65	24.42	5.49	8.14
Sichuan spicy sausage (F11)	21.16	43.03 ± 0.41	23.27 ± 0.07	6.40 ± 0.05	5.84 ± 0.10
Tian Chinese Sausage (F12)	19.85	36.83 ± 1.50	24.74 ± 4.18	6.37 ± 0.03	14.77 ± 1.30
Harbin Red Sausage (C1)	55.23	10.19 ± 0.43	14.12 ± 0.74	6.64 ± 0.03	1.72 ± 0.25
Garlic Red Sausage (C2)	59.41	15.17 ± 0.42	14.43 ± 0.29	6.50 ± 0.03	2.37 ± 0.41
Shanghai Red Sausage (C3)	65.88	3.87 ± 0.39	15.40 ± 0.41	6.50 ± 0.04	3.04 ± 0.12
Beidaihe Sausage (C4)	62.09	3.47 ± 0.25	26.72 ± 0.18	6.45 ± 0.05	1.71 ± 0.16
Fried Sausage (C5)	63.23	10.72 ± 0.24	14.07 ± 0.16	6.48 ± 0.02	4.43 ± 0.28
Tender Square Sausage (C6)	67.67	3.30 ± 1.40	11.71 ± 0.02	6.61 ± 0.05	3.33 ± 0.18
Jinluo Ham Sausage (C7)	68.90	2.87 ± 0.22	14.48 ± 0.15	6.54 ± 0.02	3.39 ± 0.67
Jinluo Chicken Sausage (C8)	63.55	5.83 ± 2.04	10.02 ± 0.03	6.88 ± 0.04	1.36 ± 0.28
Shuanghui Corn Sausage (C9)	67.12	0.89 ± 0.09	10.92 ± 0.01	6.79 ±0.03	2.28 ± 0.46
Average	63.67	6.26	14.65	6.60	2.62

Table S4 α-Dicarbonyls, CML, and CEL concentrations in sausages (mg/kg).

Samples	3-DG	DA	MGO	GO	Sum of 4 α-DCs	CML	CEL	Sum of CML and CEL
F1	0.80 ± 0.08	0.20 ± 0.01	2.14 ± 0.19	1.66 ± 0.14	4.80 ± 0.40	34.82 ± 5.96	11.17 ± 0.37	45.9 ± 6.33
F2	0.58 ± 0.11	0.18 ± 0.02	0.81 ± 0.01	1.55 ± 0.11	3.12 ± 0.17	17.89 ± 0.36	13.42 ± 1.19	31.31 ± 1.33
F3	0.69 ± 0.09	0.28 ± 0.02	2.18 ± 0.11	0.97 ± 0.08	4.12 ± 0.28	12.98 ± 1.02	14.30 ± 2.08	27.28 ± 3.03
F4	0.49 ± 0.09	0.25 ± 0.00	1.99 ± 0.09	1.06 ± 0.07	3.80 ± 0.19	20.92 ± 4.22	14.74 ± 0.84	35.67 ±3.47
F5	0.23 ± 0.04	0.26 ± 0.03	0.65 ± 0.18	1.10 ± 0.15	1.87 ± 0.83	7.38 ± 1.27	8.26 ± 0.37	15.64 ± 1.56
F6	nd	0.16 ± 0.00	1.11 ± 0.12	1.77 ± 0.11	3.05 ± 0.23	14.97 ± 0.79	10.40 ± 2.60	25.37 ± 1.93
F7	0.37 ± 0.02	0.18 ± 0.01	2.14 ± 0.08	1.36 ± 0.03	4.05 ± 0.12	21.13 ± 0.54	10.40 ± 0.29	31.54 ± 0.58
F8	nd	0.28 ± 0.00	2.57 ± 0.09	1.59 ± 0.12	4.44 ± 0.09	8.07 ± 2.23	10.69 ± 2.25	18.76 ± 4.48
F9	2.92 ± 0.21	0.76 ± 0.04	1.66 ± 0.02	1.46 ± 0.15	6.57 ± 0.59	9.38 ± 0.75	22.99 ± 1.80	32.37 ± 1.50
F10	6.93 ± 0.58	0.09 ± 0.02	0.68 ± 0.04	0.09 ± 0.06	7.79 ± 0.65	15.98 ± 2.35	21.85 ± 1.08	37.83 ± 3.20
F11	4.93 ± 0.10	0.13 ± 0.01	3.06 ± 0.11	0.21 ± 0.03	8.35 ± 0.21	12.07 ± 0.36	52.32 ± 2.34	68.24 ± 6.94
F12	0.38 ± 0.02	0.10 ± 0.01	0.43 ± 0.01	0.20 ± 0.05	1.11 ± 0.08	11.69 ± 7.71	10.83 ± 1.23	24.50 ± 4.84
Average	1.53	0.24	1.62	1.08	4.47	15.61	16.78	32.87
C1	1.39 ± 0.13	0.32 ± 0.02	2.25 ± 0.23	1.19 ± 0.11	5.15 ± 0.46	15.17 ± 0.15	13.91 ± 2.78	33.21 ± 9.89
C2	1.18 ± 0.05	0.29 ± 0.04	1.71 ± 0.08	1.19 ± 0.14	4.38 ± 0.18	8.90 ± 2.08	14.22 ± 0.89	23.12 ± 2.97

C3	0.90 ± 0.10	0.08 ± 0.00	0.21 ± 0.06	0.06 ± 0.04	1.25 ± 0.19	3.67 ± 0.31	5.89 ± 0.99	9.56 ± 1.30
C4	0.61 ± 0.07	0.16 ± 0.01	0.79 ± 0.03	0.13 ± 0.03	1.69 ± 0.14	46.11 ± 1.05	47.55 ± 3.13	96.61 ± 9.05
C5	0.60 ± 0.05	0.22 ± 0.07	1.89 ± 0.28	0.12 ± 0.05	2.84 ± 0.44	5.51 ± 0.42	17.05 ± 1.05	22.56 ± 1.05
C6	3.14 ± 0.19	0.12 ± 0.01	0.75 ± 0.08	0.05 ± 0.03	4.07 ± 0.29	6.14 ± 0.09	8.62 ± 0.15	14.76 ± 0.24
C7	0.74 ± 0.06	0.15 ± 0.02	1.50 ± 0.03	0.08 ± 0.02	2.68 ± 0.47	4.98 ± 0.42	16.20 ± 1.03	21.18 ± 1.37
C8	0.69 ± 0.05	0.15 ± 0.01	1.14 ± 0.06	0.27 ± 0.06	2.25 ± 0.17	6.76 ± 0.13	9.80 ± 0.24	16.56 ± 0.22
C9	1.29 ± 0.10	0.20 ± 0.01	1.70 ± 0.10	0.14 ± 0.04	3.33 ± 0.21	7.86 ± 0.38	15.40 ± 0.66	23.26 ± 0.90
Average	1.17	0.19	1.33	0.36	3.05	11.68	16.52	28.98

[1] nd means not detected.

Table S5 N-nitrosamines contents of collected sausages (μg/kg).

Samples	NDEA	NMEA	NDMA	NDPA	NMOR	NPIP	NPYR	NDBA	NDPhA	Sum
F1	1.61 ± 0.30	nd	8.18 ± 2.08	nd	nd	1.50 ± 0.14	nd	0.80 ± 0.14	0.08 ± 0.10	12.90 ± 3.64
F2	1.04 ± 0.23	nd	8.59 ± 1.77	nd	nd	3.79 ± 0.48	nd	nd	0.20 ± 0.11	13.02 ± 2.96
F3	1.25 ± 0.17	nd	8.05 ± 0.29	nd	nd	0.58 ± 0.06	nd	nd	0.51 ± 0.12	10.39 ± 0.46
F4	1.14 ± 0.04	nd	6.10 ± 0.24	nd	nd	1.70 ± 0.25	nd	0.62 ± 0.03	0.37 ± 0.32	9.95 ± 0.28
F5	1.59 ± 0.01	nd	6.28 ± 0.97	nd	nd	0.48 ± 0.14	nd	0.81 ± 0.12	0.13 ± 0.10	15.88 ± 1.27
F6	2.18 ± 0.27	nd	10.39 ± 1.08	nd	nd	2.05 ± 0.15	nd	1.13 ± 0.12	0.20 ± 0.19	15.63 ± 0.95

F7	2.00 ± 0.40	nd	10.52 ± 0.39	nd	nd	1.96 ± 0.13	nd	0.94 ± 0.10	0.21 ± 0.02	6.86 ± 3.73
F8	1.46 ± 1.04	nd	4.80 ± 2.90	nd	nd	nd	nd	0.39 ± 0.22	2.07 ± 0.20	9.14 ± 1.81
F9	1.35 ± 0.34	nd	5.71 ± 1.79	nd	nd	nd	nd	nd	0.16 ± 0.01	1.37 ± 0.47
F10	1.88 ± 0.01	nd	4.30 ± 0.82	nd	nd	0.27 ± 0.07	nd	0.49 ± 0.05	0.47 ± 0.37	1.36 ± 0.31
F11	1.39 ± 0.13	nd	4.25 ± 0.74	nd	nd	nd	nd	nd	0.13 ± 0.18	6.93 ± 1.65
F12	1.60 ± 0.41	nd	6.35 ± 1.39	nd	nd	0.23 ± 0.06	nd	nd	0.24 ± 0.08	8.47 ± 3.89
Average	1.54	0	6.96	0	0	1.05	0	0.37	0.40	9.32
C1	1.20 ± 0.48	nd	nd	nd	nd	nd	nd	nd	0.26 ± 0.07	4.17 ±0.88
C2	0.89 ± 0.64	nd	nd	nd	nd	nd	nd	nd	0.25 ± 0.22	7.19 ± 0.67
C3	0.89 ± 0.03	nd	5.51 ± 0.16	nd	nd	nd	nd	nd	0.50 ± 0.30	9.67 ± 1.08
C4	1.29 ± 0.70	nd	nd	nd	nd	nd	nd	nd	0.25 ± 0.08	1.35 ± 0.56
C5	2.13 ± 0.04	nd	6.09 ± 2.94	nd	nd	nd	nd	nd	0.34 ± 0.07	5.99 ± 0.88
C6	1.06 ± 0.01	nd	2.84 ± 0.68	nd	nd	nd	nd	nd	0.42 ± 0.11	8.60 ± 1.83
C7	2.53 ± 0.17	nd	5.11 ± 0.40	nd	nd	nd	nd	1.08 ± 0.04	0.46 ± 0.27	9.07 ± 0.25
C8	2.08 ± 0.19	nd	5.14 ± 0.46	nd	nd	nd	nd	nd	0.39 ± 0.08	7.61 ± 0.58
C9	1.86 ± 0.19	nd	4.31 ± 0.59	nd	nd	nd	71.73 ± 2.95	2.20 ± 0.12	0.34 ± 0.09	80.31 ± 2.80
Average	1.57	0	3.22	0	0	0	7.97	0.35	0.36	14.88

[1] nd means not detected.

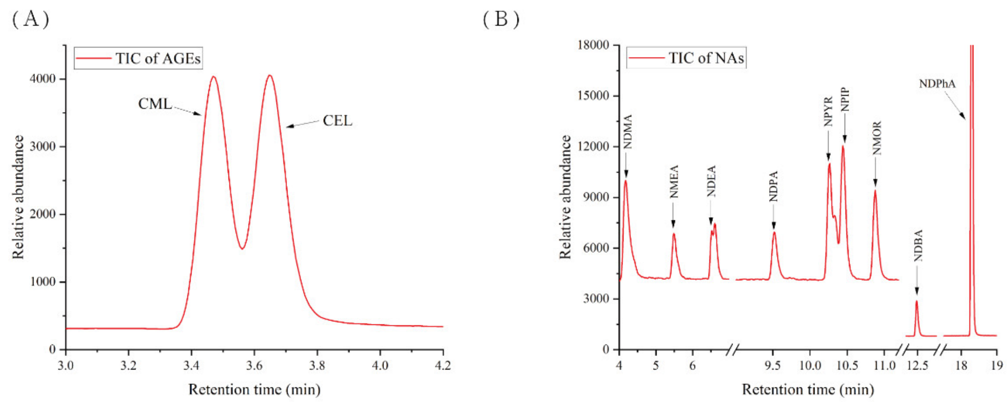

Figure S1. Representative chromatograms for AGEs and NAs. (A) TIC (Total ion chromatogram) for advanced glycation end-products, concentration: 200 ng/mL. (B) TIC for N-nitrosamines, concentration: 80 ng/mL.
